# A novel mechanism of angiotensin II-regulated placental vascular tone in the development of hypertension in preeclampsia

**DOI:** 10.18632/oncotarget.15416

**Published:** 2017-02-16

**Authors:** Qinqin Gao, Jiaqi Tang, Na Li, Xiuwen Zhou, Yongmei Li, Yanping Liu, Jue Wu, Yuxian Yang, Ruixiu Shi, Axin He, Xiang Li, Yingying Zhang, Jie Chen, Lubo Zhang, Miao Sun, Zhice Xu

**Affiliations:** ^1^ Institute for Fetology, First Hospital of Soochow University, Suzhou, China; ^2^ Center for Perinatal Biology, Loma Linda University, Loma Linda, California, USA

**Keywords:** preeclampsia, placenta vascular, angiotensin II, Pathology Section

## Abstract

The present study tested the hypothesis that angiotensin II plays a role in the regulation of placental vascular tone, which contributes to hypertension in preeclampsia. Functional and molecular assays were performed in large and micro placental and non-placental vessels from humans and animals. In human placental vessels, angiotensin II induced vasoconstrictions in 78.7% vessels in 155 tests, as referenced to KCl-induced contractions. In contrast, phenylephrine only produced contractions in 3.0% of 133 tests. In non-placental vessels, phenylephrine induced contractions in 76.0% of 67 tests, whereas angiotensin II failed to produce contractions in 75 tests. Similar results were obtained in animal placental and non-placental vessels. Compared with non-placental vessels, angiotensin II receptors and β -adrenoceptors were significantly increased in placental vessels. Compared to the vessels from normal pregnancy, angiotensin II-induced vasoconstrictions were significantly reduced in preeclamptic placentas, which was associated with a decrease in angiotensin II receptors. In addition, angiotensin II and angiotensin converting enzyme in the maternal-placenta circulation in preeclampsia were increased, whereas angiotensin I and angiotensin1-7 concentrations were unchanged. The study demonstrates a selective effect of angiotensin II in maintaining placental vessel tension, which may play an important role in development of hypertension in preeclampsia.

## INTRODUCTION

Preeclampsia is an important clinical problem, yet the etiology remains unclear. The placenta plays a critical role in the development of preeclampsia because delivery of the placenta and fetus is the only known efficient approach to resolve hypertension. Circulating factors or signals from placental ischemia or impaired placental blood flow have been considered as links between the placenta and maternal vascular dysfunction [1–6]. However, the regulatory mechanisms of placental vascular dysfunction and their contributions to the development of hypertension in preeclampsia are unclear.

Vascular dysfunction is important in the development of hypertension in various conditions, including preeclampsia. Our recent study has demonstrated that placental vascular trees behave very differently from non-placental vessels, with very limited endothelial functions, and dysfunction in vascular smooth muscle cells may play a critical role in development of hypertension in preeclampsia [7]. Placental vascular dysfunction is likely an important cause for impaired placental blood flow in preeclampsia, although physiological and pathophysiological activities of placental vessels under normal and preeclamptic conditions remain unclear. Notably, only a few previous studies investigated placental vascular functions directly in preeclampsia [8–10]. The present study was conducted in a large number of human placental and non-placental vessels from normal and preeclamptic pregnancies, as well as animal placental and non-placental vessels. We seek to reveal special features of placental vascular regulations and the pathophysiological changes under the preeclamptic condition, and to understand possible contributions of placental vascular dysfunction in the development of hypertension in preeclampsia.

## RESULTS

### Vasoconstrictions in placental *vs*. non-placental vessels

**Figure 1:**
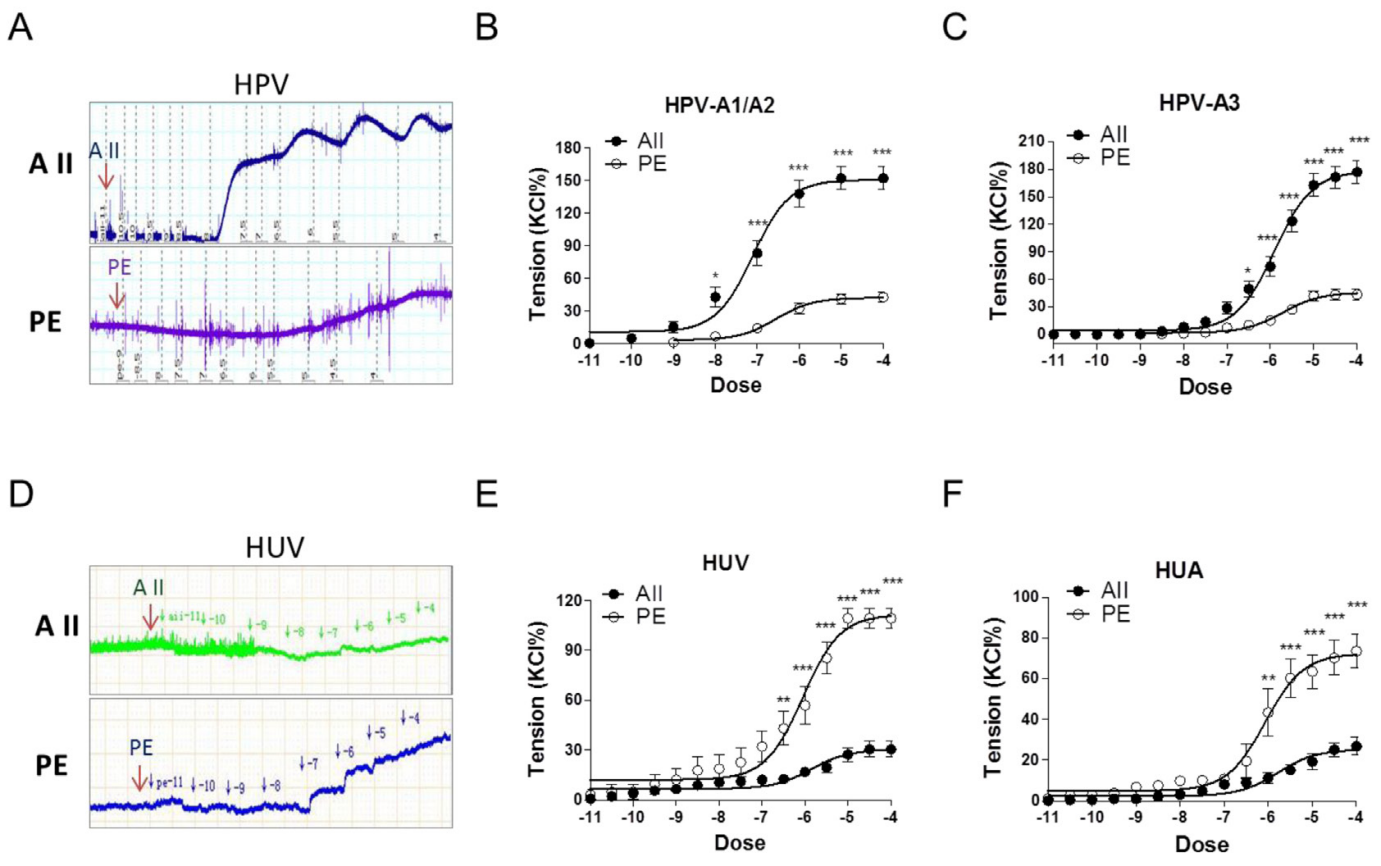
Angiotensin II and PE induced concentration-dependent vasoconstrictions in HPV, HUV, and HUA **A**. and **D**., representative images of AII- and PE-mediated dose-dependent vasoconstrictions in HPV-A3 **A**. and HUV **D**.. **B**., **C**., **E**. and **F**., AII and PE induced vasoconstrictions in HPV-A1/A2 (N = 25, *n* = 79 for AII; N = 22, *n* = 71 for PE), HPV-A3 (N = 54, *n* = 76 for AII; N = 40, *n* = 62 for PE), HUV (N = 28, *n* = 75 for AII; N = 21, *n* = 67 for PE), and HUA (N = 20, *n* = 53 for AII; N = 23, *n* = 58 for PE). AII, angiotensin II; PE, phenylephrine; HPV, human placental vessels; HUV, human umbilical vein; HUA, human umbilical artery; HPV-A1/A2, first-, second-order branch of umbilical vessels in placenta (mainly the main stem villous arteries); HPV-A3, the branch of the main stem villous arteries (micro-vessels with diameter around 150 um). Error bars denote s.e.m. *P < 0.05; **P < 0.01; ***P < 0.001. N, number of participants; n, number of rings.

Angiotensin II (AII) and catecholamines caused dose-dependent vasoconstrictions in human placental and non-placental vessels (umbilical cord vein and artery) (Figure 1A-1F; Supplementary Figure 1 and Table 1). In human placental vessels, the maximal response to AII was significantly greater than that induced by phenylephrine (PE), norepinephrine (NE), or epinephrine (E) (Figure 1A-1C, *P* < 0.05; Supplementary Figure 1, *P* < 0.05). In human umbilical vein and artery, PE-induced maximal contraction was significantly higher than that induced by AII (Figure 1D-1F, *P* < 0.05). These results demonstrated that placental vascular responses to AII were significantly more sensitive than those of the non-placental vessels in humans.

Despite the large sample size, variations due to genetic and individual factors in human experiments are always of concern. Thus, we also studied vessels from animals. Sheep placental vessels showed greater responses to AII than those induced by PE (AII > PE) (Supplementary Figure 2a, *P* < 0.05). The opposite responses (PE > AII) were observed in fetal sheep carotid, renal, and middle cerebral arteries (Supplementary Figure 2b-2d, *P* < 0.05). Furthermore, we tested rabbit carotid arteries, rat mesenteric arteries and thoracic aorta. Vasoconstriction responses to PE were also significantly greater than those induced by AII in all these non-placental vessels (Supplementary Figure 3, *P* < 0.05). Thus, vasoconstriction responses in the human and sheep placental vessels were AII > catecholamines; whereas those in human, sheep, rabbit, and rat non-placental vessels were catecholamines > AII.

### Angiotensin II- and PE-mediated vasoconstrictions in preeclamptic placental vessels

**Figure 2:**
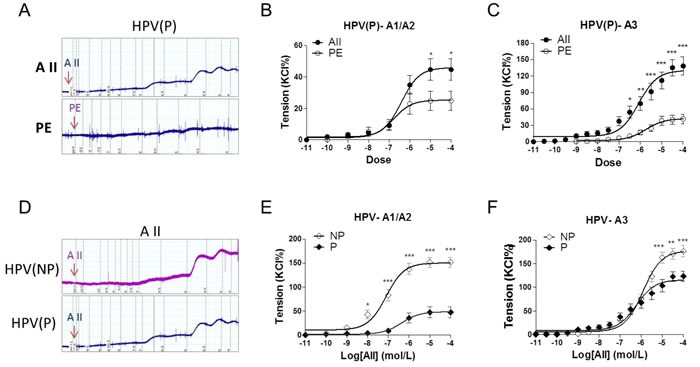
Angiotensin II and PE induced concentration-dependent vasoconstrictions in placenta vessels from normal and preeclamptic pregnancies **A**., representative images of AII- and PE-mediated dose-dependent vasoconstrictions in HPV from preeclamptic pregnancies. **B**., and **C**., AII and PE induced vasoconstrictions in HPV-A1/A2 (N = 24, *n* = 46 for AII; N = 28, *n* = 54 for PE) and HPV-A3 (N = 23, *n* = 32 for AII; N = 25, *n* = 38 for PE) from preeclampsia. **D**., representative images of AII-mediated dose-dependent vasoconstrictions in HPV from normal and preeclamptic pregnancies. **E**., and **F**., AII induced concentration-dependent vasoconstrictions in HPV-A1/A2 (N = 25, *n* = 79 for NP; N = 24, *n* = 46 for P) and HPV-A3 (N = 54, *n* = 76 for NP; N = 23, *n* = 32 for P). NP, normal pregnancy; P, preeclampsia. Error bars denote s.e.m. *P < 0.05; **P < 0.01; ***P < 0.001. N, number of participants; n, number of rings.

In preeclamptic placental vessels, the maximal response to AII was significantly higher than that induced by PE (Figure 2A-2C, *P* < 0.05). In HPV-A1/A2 (first-, second-order branch of vessels in placenta) and HPV-A3 (micro-vessels with diameter around 150 um), AII-induced maximal contractions were 151.9±10.5%, 176.5±11.7% in the normal pregnancy (NP), and 47.5±11.6%, 123.4±10.4% in preeclampsia (P), respectively (Figure 2D-2F, *P* < 0.05), showing that placental vessels in preeclampsia were less sensitive to AII.

### Expressions of AII and adrenergic receptors in placental *vs*. non-placental vessels

**Figure 3:**
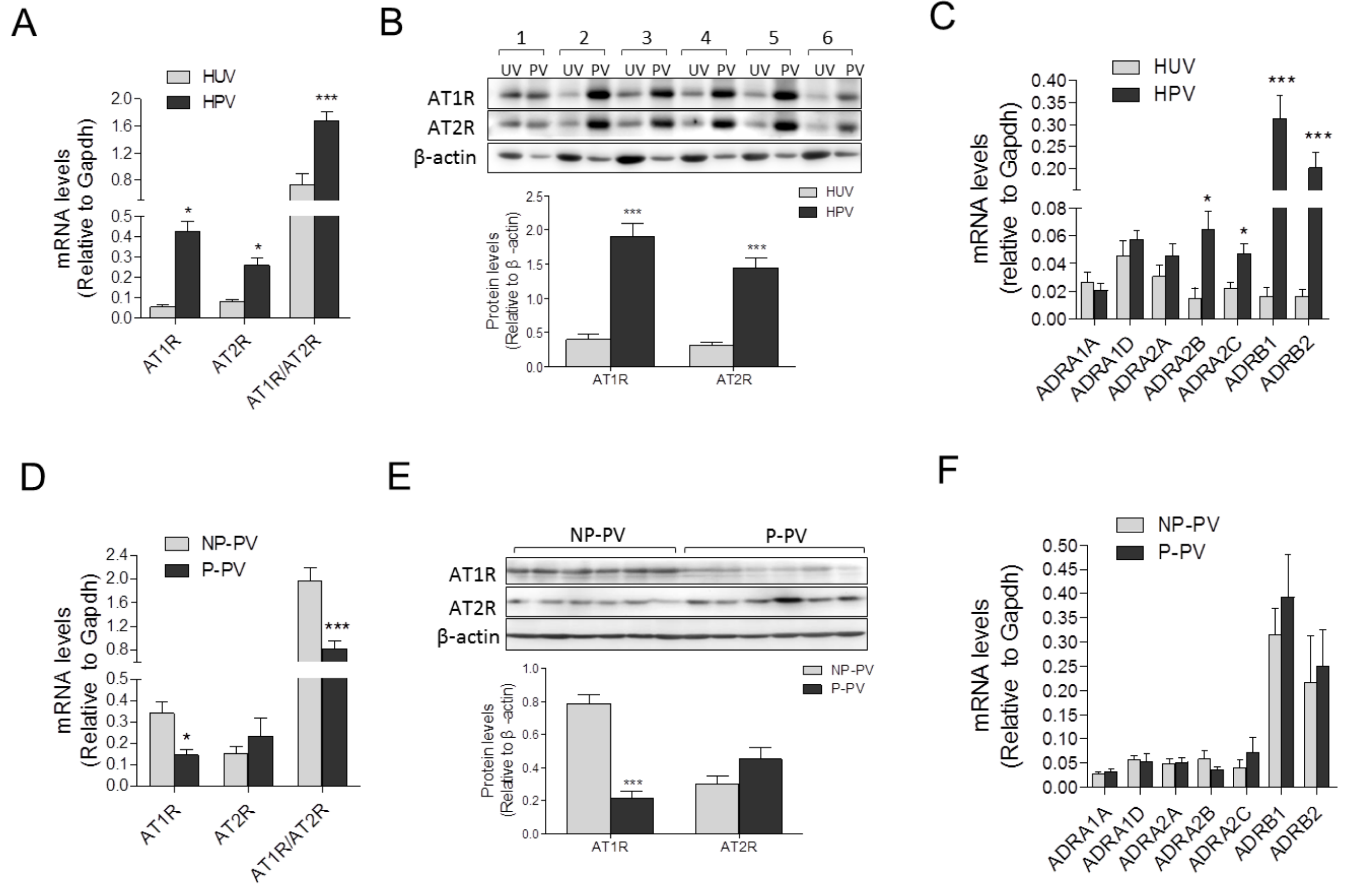
Expressions of AII and PE receptors in placental and non-placental vessels **A**., and **B**., mRNA and protein levels of both AT1R and AT2R in HPV (N = 21) and HUV (N = 18). **C**., mRNA levels of PE multifarious receptors including ADRA1A, ADRA1D, ADRA2A, ADRA2B, ADRA2C, ADRB1, and ADRB2 in HPV (N = 20) and HUV (N = 15). **D**., and **E**., mRNA and protein levels of both AT1R and AT2R in NP (N = 23) and P (N = 18) placental vessels. **F**., mRNA levels of PE multifarious receptors in NP and P placental vessels (N = 18/group). NP, normal pregnancy; P, preeclampsia. Error bars denote s.e.m. *P < 0.05; **P < 0.01; ***P < 0.001. N, number of participants.

As shown in Figure 3A and 3B, compared with human umbilical vessels, mRNA and protein abundance of AT1R, AT2R and the AT1R/AT2R ratio, were significantly increased in placental vessels. The mRNA abundance of ADRA2B, ADRA2C, ADRB1, and ADRB2 was higher in placental vessels, whereas there were no significant differences in ADRA1A, ADRA1D, and ADRA2A between placental vessels and the umbilical vein (Figure 3C). Compared with fetal sheep carotid arteries, although mRNA levels of AT1R and AT2R were not significantly changed, the AT1R/AT2R ratio was significantly increased in sheep placental vessels (Supplementary Figure 4a). There was no significant difference in mRNA abundance in ADRA1A, ADRA1B, ADRA2A, ADRA2B, ADRB2, and ADRB3 between sheep placental and non-placental vessels (carotid arteries) (Supplementary Figure 4b).

Compared with the NP, mRNA and protein levels of AT1R in preeclamptic placental vessels were significantly downregulated, leading to a pronounced decrease in the AT1R/AT2R ratio (Figure 3D and 3E). In placental vessels, the mRNA abundance of ADRA1A, ADRA1D, ADRA2A, ADRA2B, ADRA2C, ADRB1, and ADRB2 showed no significant difference between NP and P groups (Figure 3F).

### Renin angiotensin system (RAS) components and catecholamines in maternal-placenta circulation

**Figure 4:**
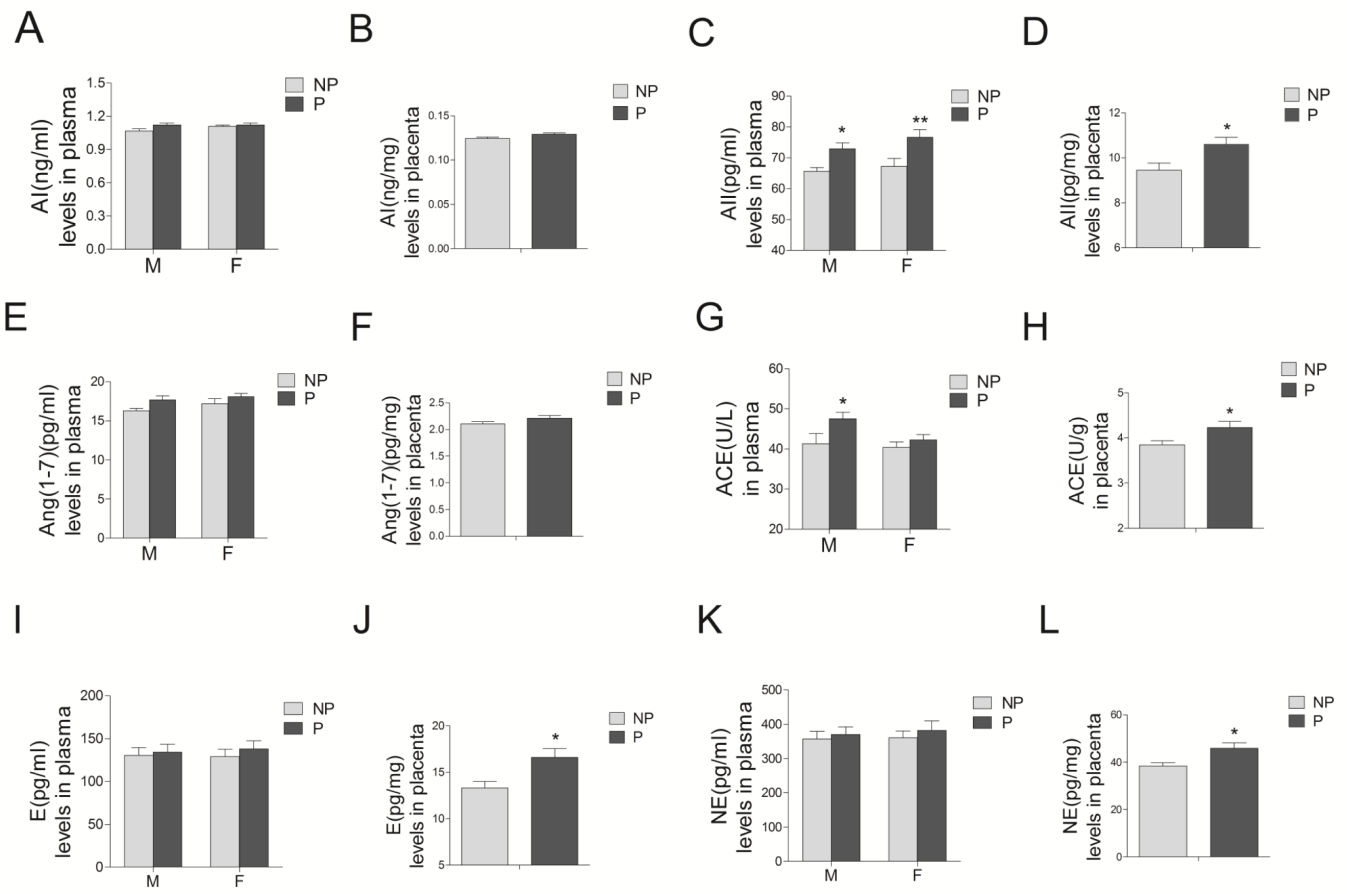
Levels of RAS components (including AI A. and B., AII C. and D., Ang1-7 E. and F., and ACE G. and H., as well as epinephrine E. I. and J.), and norepinephrine (NE) K. and L. in maternal blood M., umbilical cord blood F., and placenta from normal and preeclamptic pregnancies (N = 12/group) RAS, renin angiotensin system; AI, angiotensin I; AII, angiotensin II; Ang1-7, angiotensin1-7; ACE, angiotensin converting enzyme. NP, normal pregnancy; P, preeclampsia. Error bars denote s.e.m. *P < 0.05; **P < 0.01. N, number of participants.

Angiotensin II concentrations in preeclamptic maternal blood, umbilical cord blood, and placental tissues were significantly increased, compared with those in the normal pregnancy (Figure 4C and 4D, P < 0.05; Supplementary Table 2). Angiotensin I (AI) and angiotensin1-7 (Ang1-7) concentrations were unchanged (Figure 4A, 4B, 4E, and 4F, *P* > 0.05). Angiotensin converting enzyme (ACE) activity in both preeclamptic maternal blood and placenta were significantly increased, whereas no significant differences were observed in umbilical cord blood (Figure 4G and 4H). Concentrations of epinephrine and norepinephrine were only significantly increased in the placenta from preeclampsia, whereas no significant differences were detected in maternal and umbilical cord blood (Figure 4I-4L).

## DISCUSSION

The present study reveals several important findings: compared to non-placental vessels, vasoconstrictions induced by AII were much stronger than those by catecholamines in both human and animal placental vessels; compared to NP, placental vascular responses to AII were decreased in preeclampsia, which was associated with a decrease in AII receptors; AII and ACE were significantly increased in the maternal-placental circulation in preeclampsia. These findings provide new insights: 1) AII plays a vital role in the maintenance of vascular tension in the placenta, similar to the role of catecholamines in non-placental vessels; 2) AII-mediated physiological function in placental vessels is injured in preeclampsia; 3) the reduced AII-mediated placental vasoconstrictions may cause compensatory responses, resulting in an increase in AII and ACE in the maternal-placental circulation that may induce hypertension in preeclampsia.

As classic vasoconstrictors, AII and catecholamines play cardinal roles in the regulation of blood pressure [11–15]. In almost all peripheral blood vessels, norepinephrine is a major regulator to maintain basal vascular tension, and the norepinephrine-induced vasoconstriction is much more important than that by AII under physiological conditions [16, 17]. This was also true in our experiments on various non-placental vessels, including sheep carotid, renal, and middle cerebral arteries, rabbit carotid arteries, rat mesenteric arteries and thoracic aorta, as well as human umbilical veins and arteries. The reason we used various vessels was to prove that most of non-placental vessels from different species or organs, show the similar response pattern to AII and catecholamines. However, in human and sheep placental vessels, AII-induced vasoconstrictions were much greater than those elicited by catecholamines. Notably, in 133 tests of human placental vessels that responded to KCl, PE only produced contractions in 3.0% of vessels, whereas AII induced contractions in 78.7% of vessels. Conversely, in human umbilical veins, PE caused constrictions in 76% of 67 vessels tested, whereas AII failed to produce any contractions in 75 vessels tested. These novel findings reveal that placental vessels are fundamentally different from non-placental vessels and suggest that AII plays an important role to maintain placental blood flow in a normal “maternal-placental-circulation”. This indicates a new hypothesis for the causes of hypertension in preeclampsia: i.e., changes of AII function in placental vessels may alter the maternal-placental-circulation and placental blood flow, leading to placental ischemia and abnormal maternal blood pressure. This notion is further supported by analysis of actions of AII in preeclamptic placental vessels. Although AII-produced vascular tension was higher than that by PE regardless of small or large placental vessels, vascular responses to AII in preeclamptic placental vessels were weaker than those in the NP, indicating that physiological function of AII in placental vessels is damaged in preeclampsia.

To determine causes for the differences between placental and non-placental vessels, AII and adrenergic receptors were measured in placental and non-placental vessels (umbilical cord) from the same women. Compared with non-placental vessels, AT1R and AT2R, as well as the AT1R/AT2R ratio, were significantly increased in the placental vessels. AT1R is a major subtype mediating AII-induced vasoconstrictions, whereas AT2R may counteract AT1R [18]. The increased AT1R and/or the AT1R/AT2R ratio in placental vessels may be a cause for the stronger vasoconstrictions induced by AII in the placenta. Adrenergic receptors (ARs) include α1-ARs, α2-ARs, and β-ARs [19]. Subtypes ADRA1A, ADRA1D, ADRA2B, and ADRA2C induce contractile responses [20]. β-ARs are divided into ADRB1, ADRB2, and ADRB3 [19], and mediate vascular dilatation [20, 21]. In the present study, ADRB1 and ADRB2 were markedly higher in placental vessels than those in non-placental vessels, which could a cause for the decreased vasoconstrictions by PE in the placenta. Unchanged expression in ADRA2B, ADRA2C, ADRB1, ADRB2, ADRA1A, ADRA1D, and ADRA2A was noted between normal and preeclamptic placental vessels. AT1R was significantly downregulated, leading to a pronounced decrease in the AT1R/AT2R ratio in preeclamptic placental vessels. Although previous work showed changes of angiotensin receptors in the placenta, the studies were conducted in placental tissues but not in placental vessels along [22, 23]. The present study was the first to compare expression of AT1R and AT2R between placental and non-placental vessels in humans, and demonstrated that AT1R was downregulated in preeclamptic placental vessels.

Previous studies investigated AII receptors and other RAS components in human placental tissues [24–26]. Unchanged or altered AII levels and RAS activities in preeclampsia were reported [27–29]. These studies suggested that the over-activity of RAS was an important mechanism for preeclampsia. Although AII and its receptors may be changed in preeclampsia [24, 27, 29], it is unknown why and how they could be altered in the disease. The present study examined placental vascular functions in the same women whose blood and placental samples were used for radioimmunoassay and molecular analysis. Interestingly, levels of AII and ACE were increased in the maternal-placental-circulation in preeclampsia. Increased ACE can enhance AII concentrations [30]. Our findings provide a new insight in the development of hypertension in preeclampsia and suggest that the placenta relies heavily on RAS activities to maintain its vessel tone and local circulation. Thus, if AII-mediated vasoconstrictions become weaker, the placental circulation may suffer from reduced vascular tension and blood flow, impairing the maternal-placental-circulation. As a result, signals are sent to the maternal and the placental circulations to produce more AII and other vascular stimulators to maintain placental vessel tension, consequently resulting in maternal vascular dysfunction and hypertension.

The limitation of the present study includes that only normal animals were used due to the lack of reliable PE animal models. Despite of this, the new findings should help to explain why most hypertension in preeclampsia starts and ends in a pattern closely linked to the placenta, and provide new insights in our understanding why maternal AII or RAS components in the circulation are altered in many preeclamptic cases. These new findings should increase further the understanding of the pathophysiology of hypertension in preeclampsia, and provide new directions of investigations and treatments for pregnant hypertension.

## MATERIALS AND METHODS

### Human/animal samples

Placentas of normal pregnancy (N = 64) or preeclampsia (N = 55) were obtained from the local hospitals, Suzhou, China. Informed consent with understanding with all experiments was obtained from participants, in accordance with the Declaration of Helsinki (2013) of the World Medical Association. The study was approved by Ethics Committee of First Hospital of Soochow University (ref. no. 2011-118). Healthy pregnancies were defined as those with blood pressures < 140/90 mmHg with no significant complications. Preeclamptic patients were defined by the onset of hypertension during pregnancy (blood pressure was 140/90 mm Hg or higher, with no hypertension before) and consistent proteinuria (300 mg/day or more) (Supplementary Table 3) [2]. Maternal and umbilical cord venous blood samples were collected, following 2,500 g centrifugation at 4°C for 10 min, and stored at -80°Cuntil analyses. Umbilical cords and placentas were immediately collected after delivery. Umbilical veins and arteries as non-placental vessels and placental vessels were carefully isolated.

Pregnant sheep (gestation: 130-135 days; term, 147±3 days, N = 10) were housed in a light-controlled room with standard food and water. Under anesthesia [31], cesarean section was performed and placental vessels and fetal vessels (including fetal carotid, renal, and middle cerebral arteries) were collected. New Zeeland rabbits (15 months old, N = 6) and Sprague-Dawley rats (5 months old, N = 10) from Animal Center of Soochow University were sacrificed by intraperitoneally sodium pentobarbital (100 mg/kg; Heng Rui Medicine, Jiangsu, China). Rabbit carotid arteries, rat mesenteric arteries and thoracic aorta were collected. All experimental procedures were approved by the Institutional Animal Care Committee and in accordance with the Guide for the Care and Use of Laboratory Animals.

### Contraction studies

Rings of human, sheep, rat, rabbit vessels were prepared as described [31]. Vessel rings were given an initial tension and adjusted to that tension for 1 hour using KCl (0.12mol/L). Vasoconstrictions were induced by cumulative additions of AII, PE, NE, or E, and were normalized to the KCl-elicited contractions. Functional testing was performed as previously described [31]. Vascular functional testing was repeated and verified by third parties who were not authors. All drugs were from Sigma (St. Louis, USA).

### Quantitative real-time pCR (qRT-PCR) and western blot

Total RNA was isolated from umbilical and placental vessels using Trizol reagent (Invitrogen) according to the manufacturer's protocol. All primers used were listed in Supplementary Table 4. AT1R and AT2R protein abundance was assessed by Western blotting normalized to β-actin. Antibodies were from Santa Cruz (Santa Cruz, CA, USA). qRT-PCR and Western blot were performed as previously described [32].

### Radioimmunoassay (RIA)

Levels of RAS components [AI, AII, Ang1-7, and ACE], E, and NE in human maternal blood, umbilical cord blood, and placenta tissue were determined with RIA using the assay kits (HY-10059, HY-10059, HY-164, HY-D0036, HY-10197 and HY-100198) according to the manufacturer's instructions (Beijing Huaying Biotech Res Inst, China.). The sensitivity was 0.001 ng/ml (AI), 0.5 pg/ml (AII), 0.01 pg/ml (Ang1-7), 0.5 U/L (ACE), 0.5 pg/ml (E), and 1 pg/ml (NE), respectively. The intra-assay and inter-assay CV were 3.5-8.5% and 7.2-9.9%, respectively.

### Data analysis and statistics

Statistic analyses were performed with either *t*-test or two-way analysis of variance. Concentration-response curves of vasoconstrictions were analyzed with Graph Pad Prism 5 (Graph Pad Software, Inc., San Diego, CA, USA). Significance was accepted at *P* < 0.05. Data were expressed as the mean ± SEM.

## SUPPLEMENTARY MATERIALS FIGURES AND TABLES



## Abbreviations

NP: normal pregnancy;
P: preeclampsia;
HUV: human umbilical vein;
HUA: human umbilical artery;
HPV: human placental vessels;
RAS: renin angiotensin system;
AI: angiotensin I;
AII: angiotensin II;
Ang1-7: angiotensin1-7;
ACE: angiotensin-converting enzyme;
PE: phenylephrine;
NE: norepinephrine;
E: epinephrine.
