# Supplementary material for: A novel mechanism of angiotensin II-regulated placental vascular tone in the development of hypertension in preeclampsia
Source: Oncotarget. 2017 Feb 16;8(19):30734–41. doi: 10.18632/oncotarget.15416 (PMC5458163; doi:10.18632/oncotarget.15416)
Supplement: Supplementary file 1 [file oncotarget-08-30734-s001.pdf]

A novel mechanism of angiotensin II-regulated placental vascular tone in the development of hypertension in preeclampsia

Supplementary Material

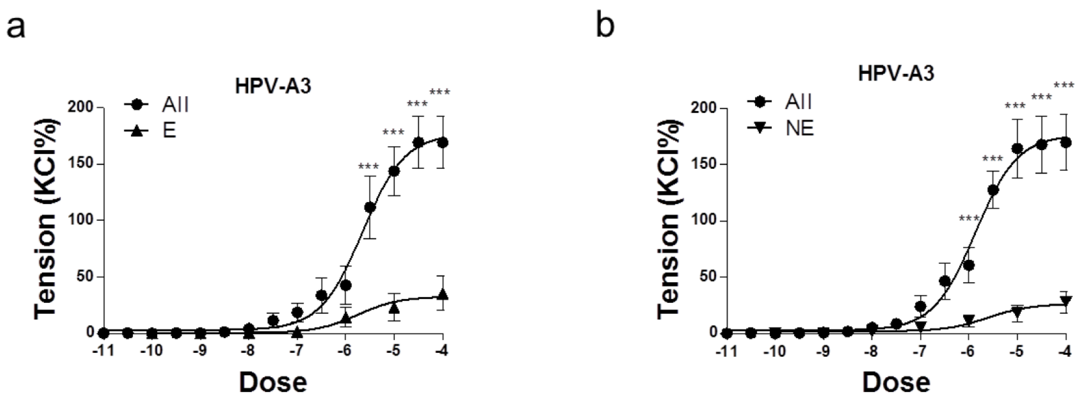

Supplementary Figure 1. Angiotensin II (AII), epinephrine (E), and norepinephrine (NE) induced concentration-dependent vasoconstrictions in human placenta micro-vessels (HPV-A3). (a), N=54, n=76 for AII; N=15, n=19 for E. (b), N=54, n=76 for AII; N=14, n=21 for NE. Error bars denote s.e.m. ***P<0.001. N, participants number; n, rings number.

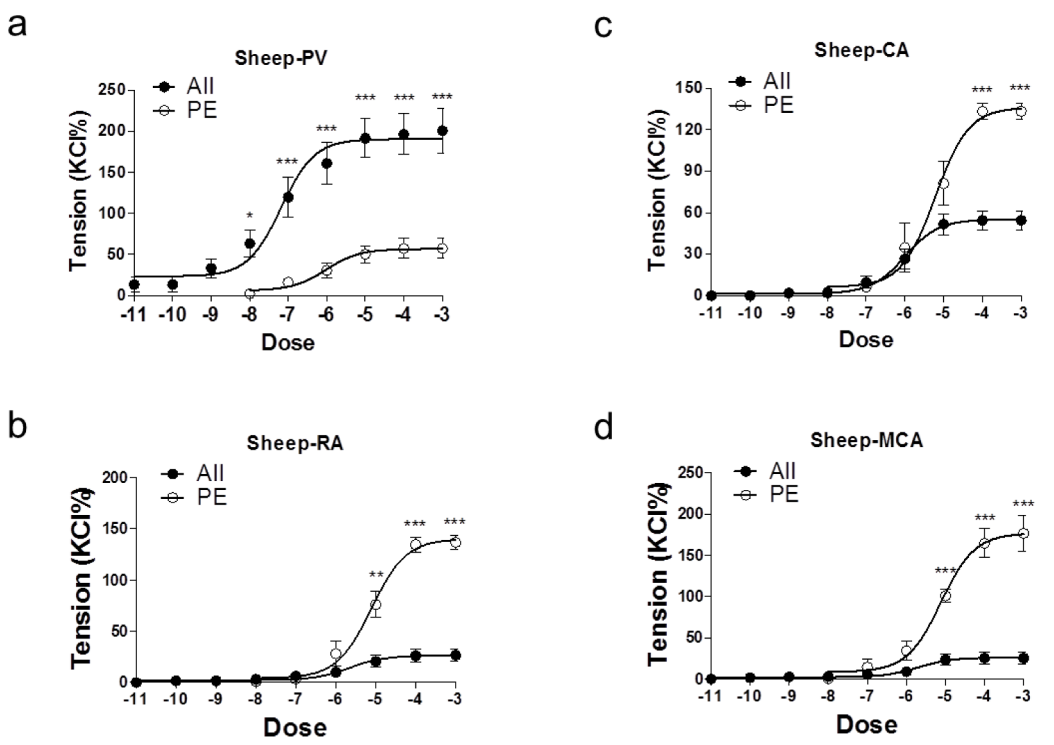

Supplementary Figure 2: The concentration-dependent vasoconstrictions induced by AII and PE in sheep placental vessels (PV) and non-placental vessels [carotid artery (CA), renal artery (RA), and middle cerebral artery (MCA)]. (a) N=10, n=23 for AII; N=9, n=17 for PE. (b) N=9, n=17 for AII; N=8, n=15 for PE; (c) N=8, n=13 for AII; N=8, n=15 for PE; (d) N=9, n=11 for AII; N=8, n=9 for PE. AII, angiotensin II; PE, phenylephrine. Error bars denote s.e.m. *P<0.05; **P<0.01; ***P<0.001. N, sheep number; n, rings number.

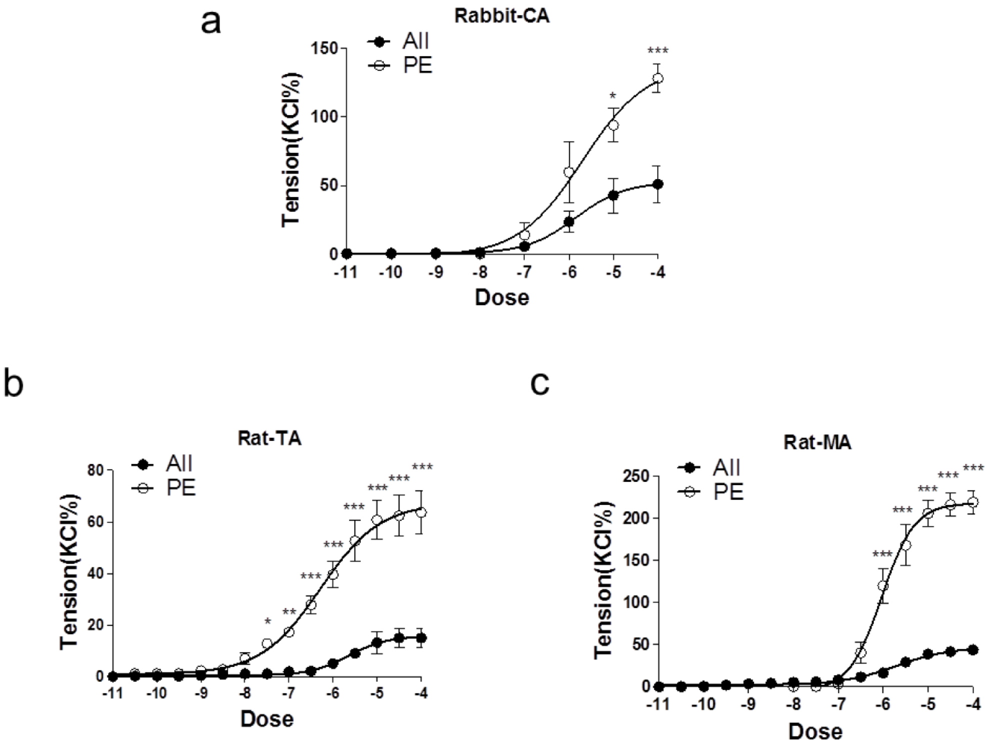

Supplementary Figure 3: The concentration-dependent vasoconstrictions induced by AII or PE in non-placental vessels. (a) rabbit carotid artery (CA), N=5, n=8 for AII; N=5, n=7 for PE; (b) rat thoracic aorta (TA), N=9, n=15 for AII; N=8, n=14 for PE; (c) rat mesenteric artery (MA) N=10, n=22 for AII; N=10, n=25 for PE. *P<0.05; AII, angiotensin II; PE, phenylephrine. Error bars denote s.e.m. **P<0.01; ***P<0.001. N, rabbit or rat number; n, rings number.

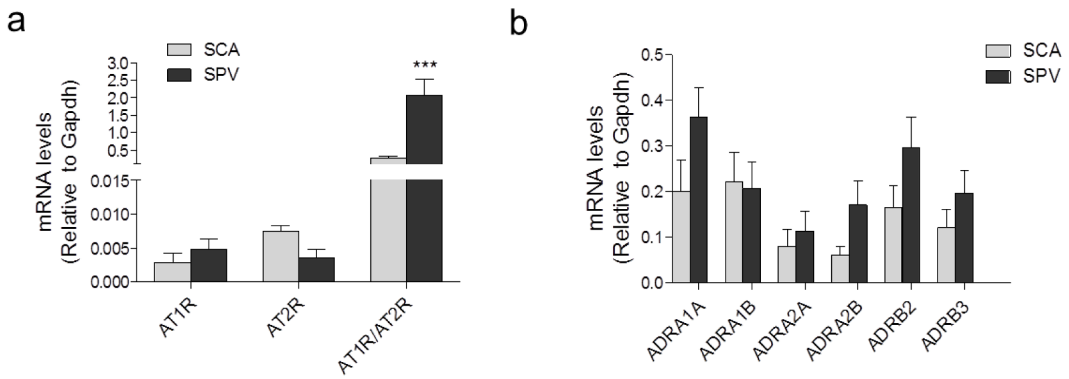

Supplementary Figure 4: The expressions of AII and PE receptors in sheep placental vessels (SPV) and non-placental vessels [carotid artery (SCA)]. (a), AT1R and AT2R; (b), ADRA1A, ADRA1B, ADRA2A, ADRA2B, ADRB2, and ADRB3. N=8-10/group. Error bars denote s.e.m. ***P<0.001. N, sheep number.

Supplementary Table 1: Percentage of vessel rings (n) for AII- or PE-induced vasoconstrictions normalized to KCl in human placenta vessels (HPV), umbilical vein (HUV), and umbilical artery (HUA). AII, angiotensin II; PE, phenylephrine. N, participants number; n, rings number.

	HPV		HUV		HUA	
	AII	PE	AII	PE	AII	PE
**N(mothers)**	64	55	28	21	20	23
**n(rings)**	155	133	75	67	53	58
**<50(KCl%)**	6(3.87%)	65(48.87%)	59(78.67%)	5(7.46%)	44(83.02%)	5(8.62%)
**100-200(KCl%)**	89(57.42%)	4(3.01%)	0	36(53.73%)	0	18(31.03%)
**>200(KCl%)**	33(21.29%)	0	0	15(22.39%)	0	0

Supplementary Table 2: Levels of renin angiotensin system components, epinephrine (E) and norepinephrine (NE) in maternal blood, umbilical cord blood, and placenta from normal and preeclamptic pregnancies. AI, angiotensin I; AII, angiotensin II; Ang1-7, angiotensin1-7; ACE, angiotensin converting enzyme. MC, maternal blood from normal pregnancy; MH, maternal blood from preeclampsia; FC, umbilical cord blood from normal pregnancy; FH, umbilical cord blood from preeclampsia; PC, placenta of normal pregnancy; PH, placenta of preeclampsia. The date was expressed as mean±SD. *P<0.05; **P<0.01. N, participants number.

	MC	MH	FC	FH		PC	PH
N	14	15	14	15		12	12
AI (ng/ml)	1.07±0.02	1.12±0.19	1.11±0.01	1.12±0.02	AI (ng/mg)	0.124±0.002	0.129±0.001
AII(pg/ml)	65.59±1.24	72.95±1.87*	67.22±2.55	76.65±1.87**	AII(pg/mg)	9.46±0.30	10.61±0.33*
ANG 1-7(pg/ml)	16.26±0.31	17.68±0.52	17.17±0.64	18.09±0.41	ANG 1-7(pg/mg)	2.10±0.04	2.21±0.05
ACE(U/L)	41.33±2.51	47.55±1.59*	41.17±1.31	42.23±1.39	ACE(U/g)	3.85±0.09	4.23±0.14*
E(pg/ml)	130.59±9.28	134.42±8.94	129.24±8.49	138.13±9.65	E(pg/mg)	13.31±0.69	16.59±0.93*
NE(pg/ml)	357.20±22.26	370.23±22.25	361.22±19.36	382.36±26.85	NE(pg/mg)	38.40±1.39	45.94±2.22*

Supplementary Table 3: Basic characteristics of normal and preeclamptic pregnancies. The date was expressed as mean±SD. *P<0.05; **P<0.01. NP, normal pregnancy; P, preeclampsia.

Characteristics	NP	P
Number of subjects	64	55
Maternal age(y)	29.30±4.30	28.95±4.45
Gestational age (wk)	37.80±2.00	34.40±5.50*
Birth weight (kg)	3.25±0.51	2.72±0.97*
Systolic BP (mm Hg)	116.8±9.20	157.5±14.70**
Diastolic BP (mm Hg)	78.30±7.30	109.2±13.30**
Proteinuria (g/24h)	0.15±0.05	7.63±4.15**
BMI--Body Mass Index	26.96±2.67	28.37±3.98

Supplementary Table 4: List of oligonucleotide primers used in this study.

qRT-PCRprimers	Sequence	
Gene Name	5'-3'	3'-5'
Human-AT1R	GCTCATCCACCAAGAAGCCT	CATTGTTCTTCGAGCAGCCG
Human-AT2R	GTGCTATTACGTCCCAGCGT	GCTTAGTGCCTAAACACACTCC
Human-ADRA1A	ACGAATAAGACAGCGCGGAA	TCGGAAGCATTTCCCGAGAG
Human-ADRA1D	CTTAGAAGGGAGAGGCTGCG	CTTGTGGGTGGTGGTAGGTC
Human-ADRA2A	ATCCTGGCCTTGGGAGAGAT	TCTCAAAGCAGGTCCGTGTC
Human-ADRA2B	AAAACCTGGCACCCAGAACA	CTCGGTGCCCTTCCAAATCT
Human-ADRA2C	CTGGATCGGCTACTGCAACA	CTCCGTCGGAAGAGGATGTG
Human-ADRB1	AATCGATCATCGTGGCTCCC	GGGTTTGCCCTACACAAGGA
Human-ADRB2	TGATCGCAGTGGATCGCTAC	GGACACGATGGAAGAGGCAA
Sheep-AT1R	AGTGAGCGCGGATTGATGTA	TAGAGGGTCCAAAGTTGTGAG
Sheep-AT2R	AAGCTGGCAAGTGTTCATAAGT	AGGGAGAAGTTGGCCTTCAT
Sheep-ADRA1A	GCCTCAAGACCGACAAGTCA	CTCACGGAGAAGTGCGTCTT
Sheep-ADRA1B	CACCCAACGATGACAGGGAA	CTCCAGCCTCCAGGTTCTTG
Sheep-ADRA2A	CCATCACCCAGGCCATAGAG	TCGAAGGAAATGAGCGGGG
Sheep-ADRA2B	GGGCAAATGGACGAGGTTTG	GGGATTCTCAGGGGAGGGAA
Sheep-ADRB2	TCACGAACCAACCCTATGCC	CTGCCTTTTGGCTACCTGGA
Sheep-ADRB3	CTGTTCCTTCAGCCTCCCAG	CTACAGTGAGGAAGCCACGG
